# Long-term effect of additional rehabilitation following botulinum toxin-A on upper limb activity in chronic stroke: the InTENSE randomised trial

**DOI:** 10.1186/s12883-022-02672-8

**Published:** 2022-04-25

**Authors:** Natasha A. Lannin, Louise Ada, Coralie English, Julie Ratcliffe, Steven Faux, Mithu Palit, Senen Gonzalez, John Olver, Emma Schneider, Maria Crotty, Ian D. Cameron

**Affiliations:** 1 Department of Neurosciences, Central Clinical School, Monash University, Melbourne, Australia; 2 Alfred Health, Melbourne, Australia; 3 School of Allied Health (Occupational Therapy), La Trobe University, Melbourne, Australia; 4 The University of Sydney, Sydney, Australia; 5 School of Health Sciences and Priority Research Centre for Stroke and Brain Injury, University of Newcastle, Newcastle, Australia; 6 College of Nursing and Health Sciences, Flinders University, Adelaide, Australia; 7 Sacred Heart Rehabilitation Unit, St Vincent’s Hospital, Sydney, Australia; 8 School of Medicine, University of New South Wales, Sydney, Australia; 9 Austin Health, Melbourne, Australia; 10 Epworth Monash Rehabilitation Medicine Research Unit, Monash University, Melbourne, Australia; 11 Rehabilitation and Aged Care, College of Medicine and Public Health, Flinders University, Adelaide, Australia; 12 John Walsh Centre for Rehabilitation Research, Northern Sydney Local Health District, Sydney, Australia

**Keywords:** Botulinum toxin type A, Stroke, Spasticity, Wrist, Rehabilitation, Neuroscience

## Abstract

**Background:**

It is common for people with persistent spasticity due to a stroke to receive an injection of botulinum toxin-A in the upper limb, however post-injection intervention varies.

**Aim:**

To determine the long-term effect of additional upper limb rehabilitation following botulinum toxin-A in chronic stroke.

**Method:**

An analysis of long-term outcomes from national, multicenter, Phase III randomised trial with concealed allocation, blinded measurement and intention-to-treat analysis was carried out. Participants were 140 stroke survivors who were scheduled to receive botulinum toxin-A in any muscle(s) that cross the wrist because of moderate to severe spasticity after a stroke greater than 3 months ago, who had completed formal rehabilitation and had no significant cognitive impairment. Experimental group received botulinum toxin-A plus 3 months of evidence-based movement training while the control group received botulinum toxin-A plus a handout of exercises. Primary outcomes were goal attainment (Goal Attainment Scale) and upper limb activity (Box and Block Test) at 12 months (ie, 9 months beyond the intervention). Secondary outcomes were spasticity, range of motion, strength, pain, burden of care, and health-related quality of life.

**Results:**

By 12 months, the experimental group scored the same as the control group on the Goal Attainment Scale (MD 0 T-score, 95% CI -5 to 5) and on the Box and Block Test (MD 0.01 blocks/s, 95% CI -0.01 to 0.03). There were no differences between groups on any secondary outcome.

**Conclusion:**

Additional intensive upper limb rehabilitation following botulinum toxin-A in chronic stroke survivors with a disabled upper limb is not more effective in the long-term.

**Trial Registration:**

ACTRN12615000616572 (12/06/2015).

**Supplementary Information:**

The online version contains supplementary material available at 10.1186/s12883-022-02672-8.

## Backgroud

Stroke represents a huge burden on the health care system. A meta-analysis has shown that botulinum toxin-A injections reduce spasticity compared to placebo [1], but that this reduction in spasticity does not carry over to an improvement in the ability to perform everyday activities [2, 3]. After formal rehabilitation ceases, it is common for people with persistent spasticity due to their stroke to attend a ‘Spasticity Clinic’ where they may receive an injection of botulinum toxin-A in the upper limb, particularly into muscles of the forearm and hand [4, 5]. Thereafter, post-injection intervention varies widely due to a lack of evidence, with around a third of Australian clinics only providing handouts or advice to encourage motor training [5] in the absence of supervised therapy. Therefore, we designed an intensive upper limb rehabilitation program based on evidence-based guidelines for stroke that was to be provided post-injection. The three-month program – InTENSE – included 2 weeks of serial casting aimed at decreasing any contracture [6] that was then followed by 10 weeks of movement training, aimed at decreasing weakness [7] and improving movement [8, 9]. The program was designed to be patient driven; it was mostly carried out at home supported by phone calls, home visits and occasional attendance at the clinic. We then conducted a Phase III randomised trial to determine the clinical effect of additional upper limb rehabilitation following botulinum toxin-A [10]. The findings suggested that, in stroke survivors attending a spasticity clinic who were scheduled to receive botulinum toxin-A to a muscle crossing the wrist, an additional 3 months of evidence-based movement training was no more effective than botulinum toxin-A plus usual care in terms of goal attainment and upper limb activity. We concluded that in chronic, severely disabled stroke survivors, it is not worthwhile spending resources on providing anything more than usual care after botulinum toxin-A. This paper presents the long-term outcomes of this Phase III clinical trial in order to see if anything had changed.

## Method

### Design

An analysis of the long-term (12 month) outcomes of the InTENSE trial was performed. The InTENSE trial was a national, multicentre, Phase III randomised trial with concealed allocation, blinded measurement and intention-to-treat analysis [11]. Stroke survivors were recruited from seven spasticity clinics across three states in Australia. Participants were randomly allocated to receive botulinum toxin-A plus evidence-based movement training or botulinum toxin-A plus usual care. Randomization was computer-generated, independent and concealed. For each clinic, allocation occurred in random permuted blocks so that after every block (of 4–8 participants), the experimental and control group contained equal numbers. Randomization occurred after injection of botulinum toxin-A. The schedule was stored off-site and group allocation was revealed online. Outcomes were measured at baseline, 3 months (end of intervention) and 12 months (9 months beyond the intervention). Measurements were collected at the clinic by researchers blind to group allocation; it was not possible to blind participants or therapists to group allocation. Data analyses were conducted by researchers blind to group allocation.

### Patients, therapists, clinics

Patients were included if they were adults over 3 months post-stroke; were scheduled to receive a botulinum toxin-A injection to a muscle(s) that crosses the wrist (in accordance with the Pharmaceutical Benefits Scheme); and were not currently receiving upper limb rehabilitation [11]. They were excluded if they had had botulinum toxin-A injections and/or casting in the past 6 months; had contraindications to botulinum toxin-A injections; had other non-stroke related upper limb conditions (e.g., fracture, frozen shoulder, arthritis); had impaired cognition (≥ 5 errors on the Short Portable Mental Status Questionnaire); or were unable to attend clinic ≥ 1/wk [11].

### Intervention

After a standard injection program according to Australian practice recommendations [12] to a muscle(s) crossing the wrist, participants in the experimental group received the InTENSE program (see TIDIER checklist [10]). This program consisted of 2 weeks of serial casting in maximum wrist extension followed by 10 weeks of movement training aimed at decreasing weakness [6] and improving active movement [8, 9] and participants were encouraged to practice for 60 min per day, 7 days a week during the 10 weeks. Participants in the control group received a handout plus one follow-up telephone call to encourage independent practice. The handout was non-individualised and contained 7 stretches, and 8 arm and hand exercises. After the 3-month period of the intervention, there was no further intervention but any botulinum toxin-A injections were recorded.

### Outcome measures

The primary outcomes were goal attainment measured using the Goal Attainment Scale [13, 14] and reported as a T-score, and upper limb activity measured using the Box and Block Test [15] and reported as blocks/s.

Secondary outcomes were spasticity, wrist extension range of motion, grip strength, pain, burden of care and quality of life. Spasticity was measured using the Tardieu Scale and reported as a score 0–4, where 0 is no spasticity [16]. Passive range of wrist extension was measured using torque-controlled goniometry and reported in degrees [17]. Grip strength was measured as a maximum voluntary contraction using a Jamar dynamometer and reported as kg [18]. Pain was measured using a visual analogue scale and reported in cm from 0–10, where 0 is no pain. Burden of care was measured using the Carer Burden Scale and reported as a score 0–16, where 0 is no burden [19]. Health-related quality of life was measured using the EuroQol-5D [20] where overall health has a value between 0 and 100, where 0 is poor health.

### Statistical analyses

Sample size was calculated to detect a between-group difference of 7 points on the Goal Attainment Scale T-score and 0.12 blocks/s on the Box and Block test with 80% power at a two-tailed significance level of 0.05. The calculation was based on the mean scores and standard deviations of the sample studied in our pilot trial [21]. On the basis of 10% attrition by 12 months, we planned to recruit a total of 136 participants, 68 per group.

An intention-to-treat analysis was conducted. Outcomes were analysed controlling for baseline values, and presented as mean between-group differences (95% CI).

## Results

### Flow of participants through the trial

140 people with stroke were recruited to the study from 03/07/2015 to 27/06/2018. Participants in both the experimental and control groups were similar in terms of their age, sex, level of education, previous living arrangements as well as chronicity, side of hemiplegia, cognition, sensation and neglect (Table 1).

**Table 1:** Characteristics of participants and centres

Characteristic	All *n* = 140	Exp *n* = 69	Con *n* = 71
Participants			
Age *(yr),* mean (SD)	61 (15)	62 (15)	60 (16)
Sex, n males (%)	97 (69)	47 (68)	50 (70)
Education, n university educated (%)	34 (24)	13 (19)	21 (30)
Living situation, n living alone (%)	24 (17)	13 (19)	11 (15)
Time since stroke (*yr*), med (IQR)	3.3 (1.6–6.2)	3.4 (1.4–6.2)	3.3 (1.6–6.2)
Side of hemiplegia, n right (%)	50 (36)	25 (36)	25 (35)
Cognition (SPMSQ, 0 to 10), mean (SD)	9.0 (1.1)	8.9 (1.2)	9.1 (1.0)
Sensation, n (%)			
Impaired	85 (61)	44 (64)	41 (58)
None	25 (18)	13 (19)	12 (17)
Neglect, n (%)			
Slight	27 (19)	13 (19)	14 (20)
Severe	4 (3)	1 (1)	3 (4)
Independent ambulation, n no (%)	78 (56)	40 (58)	38 (54)

*Exp* experimental group, *Con* control group, *SPMSQ* Short Portable Mental Status Questionnaire

**Fig. 1:**
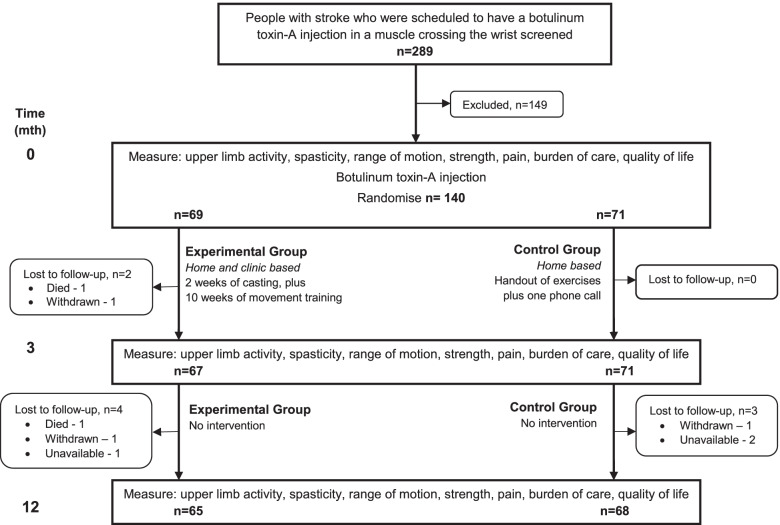
Design of and flow of participants through the study

The flow of participants through the trial is shown in Fig. 1. By Month 12, 7 participants (5%) were lost to follow-up – four from the experimental group and three from the control group. Therefore, 95% of the primary outcome – goal attainment – was collected. In addition, there was some missing data so that 93% of the other primary outcome – upper limb activity – was collected.

### Compliance with trial method

During the intervention period, both the experimental and control group spent time each day practicing motor tasks to improve their upper limb activity. The control group did about half of the amount of practice as the experimental group both at the beginning of the intervention (28 vs 52 min/day) and at the end (20 vs 37 min/day). After the cessation of intervention, 35 (25%) participants went on to have at least one more injection of botulinum toxin-A to a muscle crossing the wrist.

### Long-term effect of intervention between experimental and control groups

Group data for the two measurement occasions, within-group differences and between-group differences are presented in Table 2 for all outcome measures. In terms of goal attainment, by 12 months the experimental group scored the same (MD 0 T-score, 95% CI -5 to 5) as the control group on the Goal Attainment Scale. In terms of upper limb activity, the experimental group moved blocks at the same speed (MD 0.01 blocks/s, 95% CI -0.01 to 0.03) as the control group on the Box and Block Test. There was no difference between groups in any secondary measure. No trial-related adverse events were recorded.

**Table 2:** Mean (SD) of groups (experimental and control), mean (SD) difference within groups, and mean (95% CI) difference between groups for all outcomes

Outcome	Groups				Difference within groups		Difference between groups
	Month 0		Month 12		Month 12 minus Month 0		Month 12 minus Month 0
	Exp (*n* = 69)	Con (*n* = 71)	Exp (*n* = 65)	Con (*n* = 68)	Exp	Con	Exp minus Con
Goal attainment GAS *(T-score)*	N/A	N/A	41 (14)	41 (13)	N/A	N/A	0 (-5 to 5)
Upper limb activity BBT *(blocks/s)*	0.03 (0.09)	0.04 (0.09)	0.04 (0.12) *n* = 64	0.04 (0.10) *n* = 66	0.01 (0.05)	0.01 (0.06)	0.01 (-0.01 to 0.03)
Spasticity Tardieu Scale *(0–4)*	2.0 (0.8)	1.9 (0.8)	1.6 (0.7) *n* = 23	1.4 (0.7) *n* = 22	-0.5 (0.8)	-0.4 (0.9)	-0.1 (-0.6 to 0.4)
Wrist extension ROM *(deg)*	35 (40) *n* = 68	38 (31)	25 (40) *n* = 61	28 (33) *n* = 64	-11 (28)	-10 (29)	-1 (-11 to 9)
Grip strength MVC dynamometry *(kg)*	3.6 (4.3)	4.7 (5.9)	3.8 (4.9) *n* = 64	4.1 (4.8) *n* = 66	0.2 (3.0)	-0.1 (4.5)	0.3 (-1.1 to 1.6)
Pain 10-cm VAS *(0–10)*	1.9 (2.7)	1.9 (2.6)	1.1 (21)	1.1 (1.9)	-0.6 (2.5)	-0.9 (2.4)	0.3 (-0.6 to 1.2)
Burden of care CBS *(0–16)*	6.7 (4.3)	6.5 (3.3)	4.8 (4.0)	4.1 (3.8)	-2.0 (5.4)	-2.5 (4.4)	0.4 (-1.2 to 2.1)
Quality of life, EQ-5D							
Overall health *(0 to 100)*	65 (21)	62 (20)	67 (23)	64 (24)	3 (24)	3 (18)	-1 (-8 to 7)

*Exp* experimental group, *Con* control group, *GAS* Goal Attainment Scale, *BBT* Box and Block Test, *ROM* range of motion, *MVC* maximum voluntary contraction, *VAS* visual analogue scale, *CBS* Carer Burden Scale, *EQ-5D* EuroQual-5D

### Post-hoc analysis of long-term effect of intervention on all participants

When the experimental and control groups were combined into one group (Table 3), by 12 months there was a trend towards improvement in upper limb activity (MD 0.01 blocks/s, 95% CI 0.00 to 0.02), and a statistically significant improvement in spasticity (MD -0.4 out of 4, 95% CI -0.7 to -0.2), contracture (MD -10 deg, 95% CI -15 to -5), pain (MD -0.8 out of 10, 95% CI -1.2 to -0.3), and burden of care (MD -2.3 out of 16, 95% CI -3.1 to -1.4).

**Table 3:** Mean (SD) of times and mean (95% CI) difference between times for all participants (*n* = 133)

Characteristic	Times		Difference between times
	Month 0	Month 12	Month 12 minus Month 0
Upper limb activity *n* = 130 BBT *(blocks/s)*	0.03 (0.09)	0.04 (0.11)	0.01 (0.00 to 0.02)
Spasticity *n* = 45 Tardieu Scale *(0–4)*	2.0 (0.7)	1.5 (0.7)	-0.4 (-0.7 to -0.2)
Wrist extension ROM *(deg)*	37 (35)	27 (37)	-10 (-15 to -5)
Grip strength *n* = 130 MVC dynamometry *(kg)*	4.0 (5.1)	4.0 (4.9)	0.1 (-0.6 to 0.7)
Pain *n* = 132 10-cm VAS *(0–10)*	1.9 (2.6)	1.1 (2.0)	-0.8 (-1.2 to -0.3)
Burden of care CBS *(0–16)*	6.7 (3.9)	4.4 (3.9)	-2.3 (-3.1 to -1.4)
Quality of life, EQ-5D Overall health *(0 to 100)*	63 (21)	66 (23)	3 (-1 to 7)

*GAS* Goal Attainment Scale, *BBT* Box and Block Test, *ROM* range of motion, *MVC* maximum voluntary contraction, *VAS* visual analogue scale, *CBS* Carer Burden Scale, *EQ-5D* EuroQual-5D

### Post-hoc analysis of long-term effect of intervention between groups who did or did not receive further botulinum toxin-A

35 (25%) participants received a mean of 1.2 further botulinum toxin-A injection sessions beyond the intervention. By 12 months, they trended towards worse goal attainment (MD -5 T-score, 95% CI -10 to 1), had worse grip strength (MD -1.5 kg, 95% CI -3.0 to 0.0), but more range of motion (MD 14 deg, 95% CI 3 to 25) than those that did not receive further botulinum toxin-A (Table 4). There was no statistically significant difference between these groups in any other measure, including spasticity.

**Table 4:** Mean (SD) of groups who did (YES) and did not (NO) receive further botulinum toxin-A, mean (SD) difference within groups, and mean (95% CI) difference between groups for all outcomes

Outcome	Groups				Difference within groups		Difference between groups
	Month 0		Month 12		Month 12 minus Month 0		Month 12 minus Month 0
	YES (*n* = 35)	NO (*n* = 105)	YES (*n* = 35)	NO (*n* = 98)	YES	NO	YES minus NO
Goal attainment GAS *(T-score)*	N/A	N/A	38 (11)	42 (15)	N/A	N/A	-5 (-10 to 1)
Upper limb activity BBT *(blocks/s)*	0.03 (0.07)	0.03 (0.09)	0.03 (0.08)	0.05 (0.12)	0.00 (0.04)	0.01 (0.06)	-0.01 (-0.03 to 0.01)
Spasticity Tardieu Scale *(0–4)*	1.9 (0.8)	2.0 (0.8)	1.5 (0.7) *n* = 12	1.5 (0.7) *n* = 33	-0.3 (1.1)	-0.5 (0.8)	0.3 (-0.3 to 0.8)
Wrist extension ROM *(deg)*	29 (40)	39 (34)	30 (35)	25 (37)	0 (30)	-14 (27)	14 (3 to 25)
Grip strength MVC dynamometry *(kg)*	5.5 (5.5)	3.7 (5.0)	4.4 (5.2)	3.8 (4.7)	-1.0 (4.1)	0.5 (3.7)	-1.5 (-3.0 to 0.0)
Pain 10-cm VAS *(0–10)*	1.0 (1.8)	2.2 (2.8)	0.7 (1.2)	1.2 (2.2)	-0.4 (2.1)	-0.9 (2.6)	0.6 (-0.4 to 1.5)
Burden of care CBS *(0–16)*	6.6 (3.3)	6.6 (4.0)	4.5 (3.6)	4.4 (4.0)	-2.0 (3.8)	-2.3 (4.4)	0.3 (-1.6 to 2.2)
Quality of life, EQ-5D Overall health *(0 to 100)*	60 (22)	64 (20)	66 (24)	66 (23)	6 (24)	2 (21)	4 (-5 to 12)

*YES* had ≥ 1 injection session beyond the intervention, *NO* had no injection sessions beyond the intervention, *GAS* Goal Attainment Scale, *BBT* Box and Block Test, *ROM* range of motion, *MVC* maximum voluntary contraction, *VAS* visual analogue scale, *CBS* Carer Burden Scale, *EQ-5D* EuroQual-5D

## Discussion

This randomised trial found that, in chronic stroke survivors attending a spasticity clinic who received botulinum toxin-A to a muscle crossing the wrist, an additional 3 months of evidence-based movement training was no more effective in the long-term than botulinum toxin-A plus a handout of exercises in terms of goal attainment and upper limb activity. When the experimental and control groups were combined, overall, there were small improvements in spasticity, contracture, pain, and burden of care. When the cohort was divided according to further botulinum toxin-A injections, those that received further botulinum toxin-A had worse grip strength but better range of motion than those that did not receive it.

In terms of the effect of the intervention as an adjunct to botulinum toxin-A, immediately after the intervention at 3 months, there was no effect on any outcome except grip strength but this effect had disappeared by 12 months so that there was no between-group difference on any outcome. The lack of effect at 12 months is not surprising in light of the lack of effect at 3 months. In addition, the participants in this trial were representative of stroke survivors attending spasticity clinics in Australia in that they were chronic and severely disabled [10] and therefore may have had a limited potential for recovery [22]. There have been 3 systematic reviews of adjunct interventions for botulinum toxin-A published recently [2, 23, 24] but none have performed a meta-analysis and none of the randomized trials included in the reviews were published after our trial began. Our trial adds to the evidence that casting increases range of motion in the short-term [25] but that movement training does not enhance the effect of botulinum toxin-A in terms of activity.

When the cohort was considered together, immediately after the intervention at 3 months and beyond the intervention at 12 months, there was a half-point reduction in spasticity, small improvements in grip strength, pain and burden of care but no changes in upper limb activity or quality of life. However, at 3 months there was an increase in range of motion (8 deg) but this had changed to a decrease (10 deg) at 12 months. This is in line with two recent systematic reviews with meta-analyses investigating botulinum toxin-A after stroke [26, 27] which both found robust evidence for a decrease in spasticity and burden of care but no increase in upper limb activity, either in the short- [26, 27] or long-term [26]. Importantly, the review by Andringa [26] claims that the evidence is robust enough not to need further trials investigating the efficacy of botulinum toxin-A, but that fully powered trials of adjunct interventions are still needed.

The decrease in range of motion at the wrist from 3 to 12 months led us to perform a post-hoc analysis differentiating those who received further botulinum toxin-A injections from those that did not. On average, 25% of stroke survivors received further botulinum toxin-A over 1.1 sessions. At 12 months, while there was no difference in spasticity between these groups, those who received further botulinum toxin-A did demonstrate increased range of wrist extension but weaker muscle strength. This is in line with an observational study of multiple injections of botulinum toxin-A [28] which concluded that multiple injections of botulinum toxin formulation may be more effective in increasing range of motion than a single injection. Taken together these findings suggest that botulinum toxin-A needs to be ongoing to be of any benefit. However, further research is required to understand whether the improvement in range of motion is sufficient to balance out the potential reduction in grip strength.

This trial has both strengths and weaknesses. Its main strength was that it was fully powered; the confidence intervals for both the BBT and GAS did not cross any worthwhile effect. Its main weakness was that the participants, while representative of those attending spasticity clinics around Australia, were some 3 years after their stroke and very disabled [10], which means that they may not have been able to make improvements in upper limb activity. Also, there were missing data for the spasticity measure at 12 months, however, the missing data was random and the 12-month findings in line with the 3-month findings. Only a small number of participants continued botulinum toxin therapy after the 3-month assessment (25%), therefore we acknowledge that future clinical trials may provide more robust data regarding the effects of repeat injections.

Measurement of goal attainment using GAS may be considered both a strength and limitation. Using GAS offered an effective method for assessing changes in specific functional domains pertaining to upper limb motor training from the participant’s perspective of what was valued. These data are, however, based on participant report. Obtaining consumer reflections of the perceived impact of the InTENSE therapy program was critical, and while a strength, it should be acknowledged that these data are unblinded. To address this potential bias, the goal interviews were completed by an assessor blind to group allocation and interpreted alongside the blinded assessment of motor function (BBT).

The Australian, Canadian, and UK guidelines [29–31] all recommend that the use of botulinum toxin-A for spasticity management should be combined with concurrent rehabilitation. However, this recommendation is based on expert opinion rather than evidence. Our fully powered clinical trial taken together with recent evidence suggests clinicians who use botulinum toxin-A to manage spasticity in chronic, disabled patients can expect an improvement in secondary impairments but should not expect an improvement at the activity level even with training and can only expect improvements to last long-term if botulinum toxin-A is ongoing.

## Supplementary Information

**Additional file 1:** 

## Data Availability

The data that support the findings of this study are available from the corresponding author on reasonable request.
