# Supplementary material for: Long-term effect of additional rehabilitation following botulinum toxin-A on upper limb activity in chronic stroke: the InTENSE randomised trial
Source: BMC Neurol. 2022 Apr 25;22:154. doi: 10.1186/s12883-022-02672-8 (PMC9036685; doi:10.1186/s12883-022-02672-8)
Supplement: Supplementary file 1 — Additional file 1: [file 12883_2022_2672_MOESM1_ESM.pdf]

Online-only Supplementary Data

Supplementary Table: Summary of initial doses of botulinum toxin-A delivered in the InTENSE Trial

Botulinum toxin-A injections	Flexor Carpi Radialis		Flexor Carpi Ulnaris		Flexor Digitorum Superficialis		Flexor Digitorum Profundis		Flexor Pollicus Longus		Extensor Carpi Radialis Longus	
	Exp	Con	Exp	Con	Exp	Con	Exp	Con	Exp	Con	Exp	Con
Participants, n (%)	44 (65)	39 (55)	50 (74)	52 (73)	60 (88)	58 (82)	39 (57)	37 (52)	25 (37)	23 (32)	5 (7)	4 (6)
Units, mean (SD)	49 (19)	47 (16)	48 (19)	50 (18)	52 (18)	53 (21)	47 (15)	51 (20)	32 (21)	38 (22)	34 (22)	20 (14)
